# Guideline-concordant chemotherapy in patients with hormone receptor-positive and node-positive, early breast cancer leads to better overall and metastases-free survival with limited benefit in elderly patients

**DOI:** 10.1007/s00404-019-05387-3

**Published:** 2019-11-20

**Authors:** Clara Taubenhansl, Olaf Ortmann, Michael Gerken, Elisabeth C. Inwald, Monika Klinkhammer-Schalke

**Affiliations:** 1 Faculty of Medicine, University of Regensburg, Universitaetsstraße 31, 93053 Regensburg, Germany; 2 Department of Gynecology and Obstetrics, University Medical Center Regensburg, Landshuter Straße 65, 93053 Regensburg, Germany; 3 Tumor Center, Institute for Quality Assurance and Health Services Research, University of Regensburg, Am BioPark 9, 93053 Regensburg, Germany

**Keywords:** Breast cancer, Hormone and node positive, Chemotherapy, Elderly patients, Overall survival, Distant metastases-free survival

## Abstract

**Purpose:**

The German guideline for breast cancer recommends using chemotherapy (CHT) in patients with hormone receptor-positive and node-positive, invasive breast cancer. The aim of this study was to analyse the effects of CHT in this patient group on overall survival (OS) and distant metastases-free survival (DMFS), especially considering the 70-year threshold.

**Methods:**

1772 patients from the clinical cancer registry Regensburg (Germany) with hormone receptor-positive and node-positive, invasive breast cancer diagnosed between 2003 and 2013 were analysed in a retrospective cohort study. OS and DMFS were evaluated by means of Kaplan–Meier and multivariable Cox-regression method. Results were further examined according to age at diagnosis.

**Results:**

The comparison of 1544 patients with CHT to 228 patients without CHT showed a significant benefit for CHT regarding 5-year OS (91.3% vs. 76.8%) and 5-year DMFS (86.7% vs. 74.4%, both *p* < 0.001). Likewise, better OS and DMFS were seen in patients aged < 70 years using CHT compared to patients without CHT of the same age. Patients aged ≥ 70 years with CHT had a minimal benefit regarding 5-year OS compared to patients without CHT, but no advantage considering DMFS. All results were confirmed in multivariable analyses except for patients being ≥ 70 years of age.

**Conclusion:**

Patients with hormone receptor-positive and node-positive, invasive breast cancer benefit from chemotherapy with regard to a significantly better overall and distant metastases-free survival, although chemotherapy use in patients aged ≥ 70 years results in a smaller benefit considering OS and no benefit considering DMFS.

## Introduction

Breast cancer is the most common cancer in women worldwide and the most frequent cause of death from cancer across all tumor types [1]. 69,870 new cases of illness and 17,804 breast cancer deaths were reported in Germany in 2014 [2]. In Germany, the interdisciplinary S3 guideline for diagnosis, treatment and aftercare of breast cancer supports the medical decision depending on the different therapy options. According to the recommendation of the S3 guideline, chemotherapy is indicated for patients with targeted therapy required, triple negative tumors or patients with a high-risk tumor type [3]. To examine the benefit and the effect of the guideline recommendation in a patient`s subgroup, this study investigates the benefit of chemotherapy treatment in patients with hormone receptor positive and node positive breast cancer. The positive effect of chemotherapy is proven in lots of studies. As a leading study, the Early Breast Cancer Trialists’ Collaborative Group (EBCTCG) documented statistically significant positive effects of adjuvant chemotherapy in reducing breast cancer recurrence and mortality, above all regarding patients being 50 years of age or less. A benefit is also established in elderly patients [4].

Nevertheless, chemotherapy treatment can be attendant on different negative short-term or long-term side effects, which have an impact on health-related quality of life [5]. Especially, elderly patients are predestined to suffer from negative side effects. These well-known consequences lead to an undertreatment of elder patients concerning the systemic therapy compared to younger patients [6]. In addition to that, there is a lack of evidence for breast cancer care in elderly patients, because age is often a reason for exclusion from randomized clinical trials on breast cancer treatment [7]. The result of which is that the use of chemotherapy should be weighed with care and is often a heavily debated topic especially in elderly breast cancer patients.

The diagnosis, therapy and follow-up care of breast cancer patients require a multi-disciplinary concept. The German Cancer Society has developed a certification program to offer patients a treatment that is based on high-quality standards. The entire certification system is organised by OnkoZert, an institution specialised in medical certification. The quality of the patient-centred care in different sectors is measured by quality indicators derived from the German guidelines that define the treatment of breast cancer patients. A reevaluation of the quality indicators is performed by yearly audits. Indicator no. 6 demands the chemotherapy treatment in patients with hormone receptor positive and node positive invasive breast cancer and has a minimum quote of 60% [8]. The intention of the present study is the analysis of this selected quality indicator of the German Cancer Society.

To our knowledge, studies with focus on distant metastases recurrence rate and distant metastases-free survival are not available.

The aim of this study was to analyse the effects of chemotherapy for patients with hormone receptor positive and node positive breast cancer on overall and distant metastases-free survival, especially considering the 70-year threshold.

## Material and methods

### Database

The current study is based on data from the Tumor Centre Regensburg (Bavaria, Germany). It is a high-quality population-based regional cancer registry collecting information about all cancer sites from Upper Palatinate and Lower Bavaria. This area comprises a population of more than 2.3 million people. The documentation includes information about diagnosis, therapies, course of disease, and long-term follow-up. The Tumor Centre obtains information about patients from the University Hospital Regensburg, 53 regional hospitals and more than 1500 practicing doctors. Medical reports, pathology, and follow-up records are the basis for the documentation in the cancer registry. The cancer registry is additionally informed by the regional registry offices and health offices about mortality data.

About 80% of all breast cancer patients are treated in specialized breast cancer centres. These institutions are focused on breast cancer and are certified by the German Cancer Society (Deutsche Krebsgesellschaft, DKG). In the mentioned area, eight breast cancer centres are included complying with the conditions the DKG claims for, i.e., standardized procedures in diagnosis, therapies and documentation. The current study is based on data from these eight breast cancer centres.

In the following paragraph, some definitions are given regarding the classifications of breast cancer subtypes used in the analyses. Concerning the nodal status, N1 is defined as 1–3, N2 as 4–9, and N3 as 10 or more affected axillary lymph nodes. Her2 is a member of the human epidermal growth factor receptor family. Her2-positive breast cancer type has an amplification or overexpression of this oncogene. The overexpression is analysed by immunohistochemistry (IHC) and fluorescence in situ hybridization (FISH). If the IHC result is 0 or 1+, the cancer is considered HER2 negative, if it is 3+, the cancer is HER2 positive. In case of an equivocal IHC result 2+, the HER2 status of the tumor needs to be tested with FISH to clarify the result. Triple negative breast cancer is defined as a missing expression of the genes for estrogen receptor (ER), progesterone receptor (PR) and HER2. The molecular subtype of breast cancer with positive hormone receptor status, Her2 negative expression, grading G1 or G2, and Ki67 expression ≤ 15% is called Luminal A. Luminal B describes the molecular subtype of breast cancer patients with positive hormone receptor status, Her2-negative expression, grading G1, G2 or G3 and Ki67 expression > 15%.

### Inclusion and exclusion criteria

**Fig. 1:**
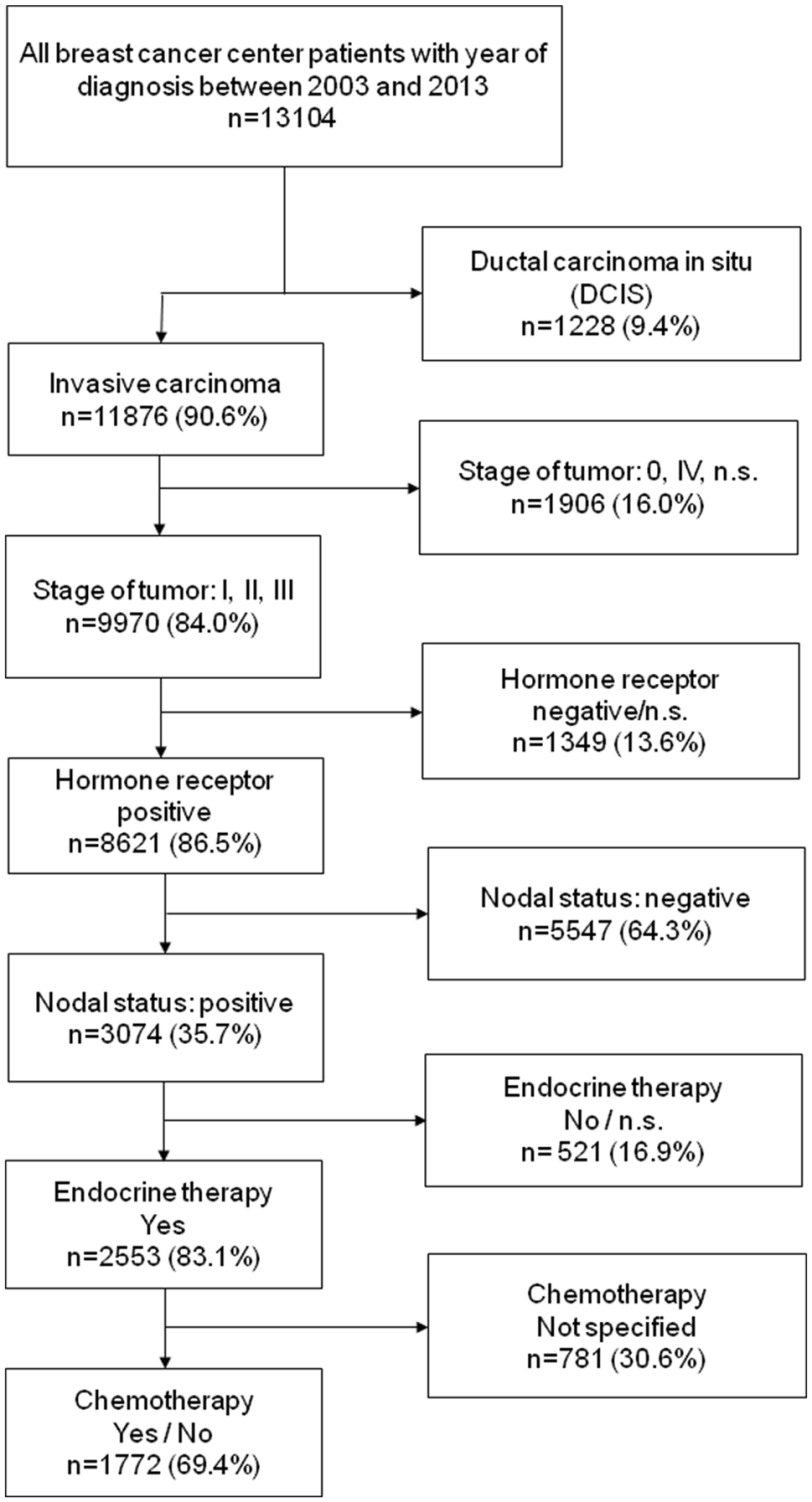
Scheme of data extraction

The present data pool is based on 13,104 breast cancer patients from Upper Palatinate and Lower Bavaria, who had been diagnosed between January 2003 and December 2013 and treated in certified breast cancer centres. To focus only on invasive breast cancer, patients with non-invasive breast cancer or primary metastatic breast cancer were excluded. Also, patients with hormone receptor-negative and node-negative tumors were omitted. To create a data pool with similar initial basis, only breast cancer patients treated with endocrine therapy were analysed. 30.6% of these patients were characterized with missing information about receiving chemotherapy or not. To achieve a precise evidence to the influence of the chemotherapy, only proven cases of realized or non-realized chemotherapy were included in the further evaluation. The inclusion criteria lead to a data pool of 1772 eligible patients (Fig. 1).

### Statistical analyses

Continuous data are expressed as means ± standard deviations (SD) and categorical data as frequency counts (percentage). Student’s *t* test for normally distributed continuous variables and Pearson’s chi-square tests for categorical variables were used for comparing the baseline characteristics of patients. Overall survival was calculated from the date of breast cancer diagnosis to the date of death from any cause. In case of recurrence-free survival rates, the first recurrence was included as event. Cases were declared as censored if there occurred no death or recurrence in the period of observation or until end of follow-up (2003–2013). Kaplan–Meier plots illustrate the overall survival, the metastases-free survival and the cumulative distant metastases recurrence rate. Cox regression models were calculated to render hazard ratios (HR) and corresponding 95% confidence intervals (95% CI). They are adjusted for the known confounding variables: age at diagnosis, grading, tumor size, nodal status, lymphatic invasion, venous invasion, and Her2 status. *p* value from Log-rank test of 0.05 was considered the threshold of statistical significance and all reported *p* values were two sided. All results were calculated with the software IBM SPSS Statistics 24.0.

## Results

### Patients’ characteristics

In total, 1544 breast cancer patients received chemotherapy (87.1%) and 228 received no chemotherapy (12.9%). On average, the percentage of patients treated with CHT decreased over the years from 97.9% in 2003 to 79.2% in 2013 (Table 1).

**Table 1:** Share of chemotherapy treatment in hormone- and node-positive breast cancer patients between 2004 and 2013

Year of diagnosis	CHT *N* (%)	No CHT *N* (%)	Total *N* (%)
2004	141(97.9%)	3 (2.1%)	144 (100.0%)
2005	158 (98.1%)	3 (1.9%)	161 (100.0%)
2006	140 (89.7%)	16 (10.3%)	156 (100.0%)
2007	143 (89.4%)	17 (10.6%)	160 (100.0%)
2008	146 (84.9%)	26 (15.1%)	172 (100.0%)
2009	168 (86.2%)	27 (13.8%)	195 (100.0%)
2010	171 (81.8%)	38 (18.2%)	209 (100.0%)
2011	168 (88.9%)	21 (11.1%)	189 (100.0%)
2012	172 (80.8%)	41 (19.2%)	213 (100.0%)
2013	137 (79.2%)	36 (20.8%)	173 (100.0%)
Total	1544 (87.1%)	228 (12.9%)	1772 (100.0%)

The distribution of the age at diagnosis was different between patients treated with CHT compared to patients without CHT (*p* < 0.001, Table 2). The majority of patients receiving CHT was between 50 and 69 years old (55.4%). In contrast, the majority of patients without CHT was 70 years of age or older (59.6%). Distribution of grading was similar in the compared groups concerning G2, which was the most diagnosed type of grading (68.9% in CHT and 68.0% in no CHT). In the CHT-treated group, the low grading type G1 was listed more rarely than the advanced grading type G3 (6.7% vs. 24.4%). The untreated group showed a smaller difference in the distribution of grading between G1 and G3 (13.2% vs. 18.9%, *p* = 0.001). Patients not obtaining CHT had more frequently a low nodal status N1 (*p* = 0.001). Additionally, this group of patients had more often no lymphatic invasion (37.3% in no CHT vs. 24.9% in CHT, *p* < 0.001). Detailed description of the data pool is shown in Table 2.

**Table 2:** Patient and tumor characteristics compared between breast cancer patients with use or with non-use of chemotherapy

	CHT		No CHT		Total		Chi^2^
	*N*	%	*N*	%	*N*	%	*p*
Age at diagnosis							
< 50	499	32.3	21	9.2	520	29.3	
50–69	855	55.4	71	31.1	926	52.3	< 0.001
≥ 70	190	12.3	136	59.6	326	18.4	
Grading							
G1	103	6.7	30	13.2	133	7.5	
G2	1064	68.9	155	68.0	1219	68.8	0.001
G3	377	24.4	43	18.9	420	23.7	
Tumor size							
T1	591	38.3	82	36.0	673	38.0	
T2	753	48.8	107	46.9	860	48.5	
T3	135	8.7	22	9.6	157	8.9	0.163
T4	65	4.2	17	7.5	82	4.6	
Nodal status							
N1	916	59.3	161	70.6	1077	60.8	
N2	393	25.5	33	14.5	426	24.0	0.001
N3	235	15.2	34	14.9	269	15.2	
Stage of tumor							
I	16	1.0	7	3.1	23	1.3	
II	844	54.7	135	59.2	979	55.2	0.011
III	684	44.3	86	37.7	770	43.5	
Lymphatic invasion							
L0	385	24.9	85	37.3	470	26.5	
L1	905	58.6	125	54.8	1030	58.1	< 0.001
L n.s	254	16.5	18	7.9	272	15.3	
Venous invasion							
V0	1050	68.0	184	80.7	1234	69.6	
V1	142	9.2	21	9.2	163	9.2	< 0.001
V n.s	352	22.8	23	10.1	375	21.2	
Her2 status							
Negative	1249	80.9	202	88.6	1451	81.9	
Positive	293	19.0	26	11.4	319	18.0	0.018
n.s	2	0.1	0	0.0	2	0.1	
Residual tumor							
R0	1488	96.4	207	90.8	1695	95.7	
R1/2	29	1.9	8	3.5	37	2.1	< 0.001
RX/n.s	27	1.7	13	5.7	40	2.3	
Total	1544	100.0	228	100.0	1772	100.0	

*n.s.* not specified

### Survival analyses

**Fig. 2:**
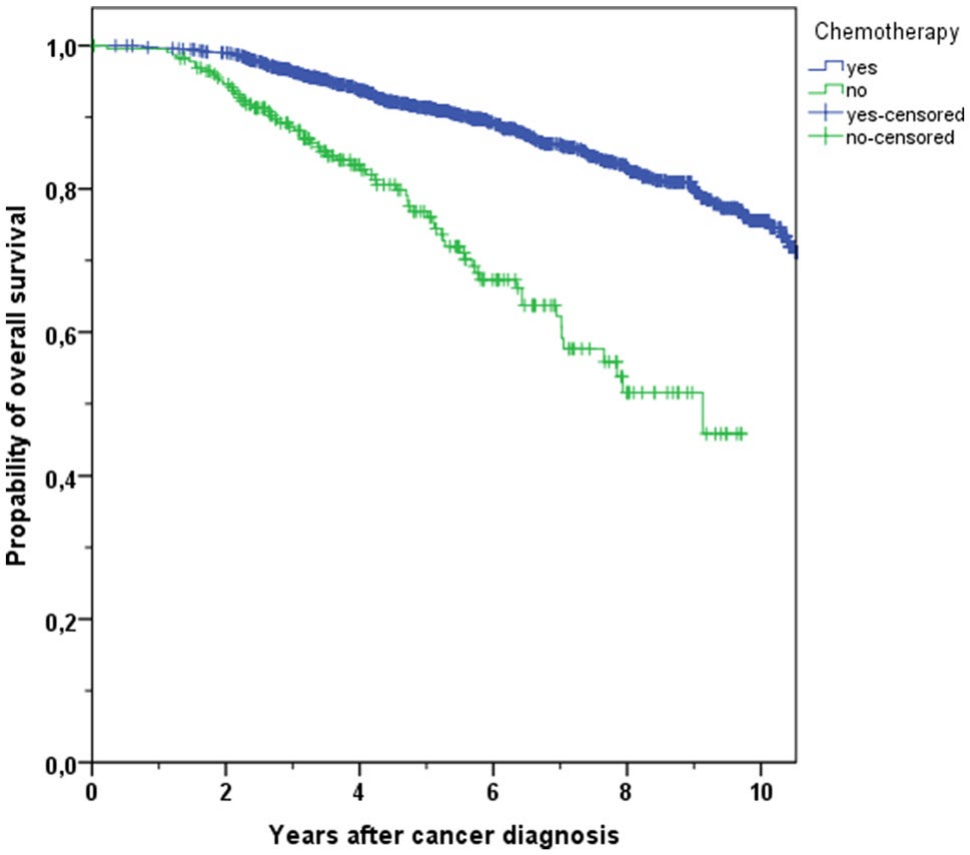
Kaplan–Meier plot of overall survival in hormone- and node-positive breast cancer patients with use or with non-use of chemotherapy

To evaluate the long-term effects of CHT, patients with or without treatment were compared considering overall survival. Mean follow-up was 6.6 years (median 6.4 years). 1544 patients were treated with CHT, 228 patients were not treated. Patients receiving CHT showed a better OS than patients without CHT (Fig. 2). In the course of the years, the difference between the survival rates increased steadily (3-year OS 96.3% vs. 88.7% and 5-year OS 91.3% vs. 76.8%, 10-year OS 85.8% vs. 72.9%, *p* < 0.001).

Adjusted to all influential variables (age of diagnosis, grading, tumor size, nodal status, lymphatic invasion, venous invasion, HER2 status) in a multivariable Cox regression model, the better OS in the CHT group was still evident (HR 0.494, 95% CI 0.343–0.711, *p* < 0.001, Table 3). Stage of tumor was rejected by the model due to collinearity with tumor size T and nodal status N. Besides CHT, age, grading, tumor size and nodal status proved to be independent factors for OS.

**Table 3:** Results of multivariable Cox proportional hazard model on overall survival

	Hazard ratio	95% CI	*p*
Chemotherapy			
No CHT	Reference		
CHT	0.494	0.343–0.711	< 0.001
Age at diagnosis			
< 40	Reference		
40–49	0.492	0.283–0.857	0.012
50–59	0.519	0.302–0.893	0.018
60–69	0.981	0.598–1.607	0.938
70–79	1.273	0.758–2.138	0.361
> 80	2.231	1.056–4.713	0.035
Grading			
G1	Reference		
G2	2.364	1.039–5.380	0.040
G3	3.280	1.413–7.614	0.006
Tumor size			
T1	Reference		
T2	1.511	1.114–2.050	0.008
T3	1.591	1.039–2.435	0.032
T4	2.627	1.655–4.172	< 0.001
Nodal status			
N1	Reference		
N2	1.360	1.007–1.837	0.045
N3	2.438	1.810–3.283	< 0.001
Lymphatic invasion			
L0	Reference		
L1	1.348	0.949–1.914	0.096
n.s	1.878	1.010–3.494	0.047
Venous invasion			
V0	Reference		
V1	1.223	0.855–1.748	0.270
n.s	0.830	0.507–1.359	0.459
Her2 status			
Negative	Reference		
Positive	1.109	0.826–1.488	0.492
n.s	7.015	0.948–51.904	0.056

*n.s.* not specified

### Distant metastases recurrence rate and distant metastases-free survival

To investigate the cumulative relapse rate of distant metastases in our study, we focussed on operated patients with R0-resection only (*N* = 1695, 95.7%). In this case, relapse includes only distant metastases, no local or lymphatic node relapse.

**Fig. 3:**
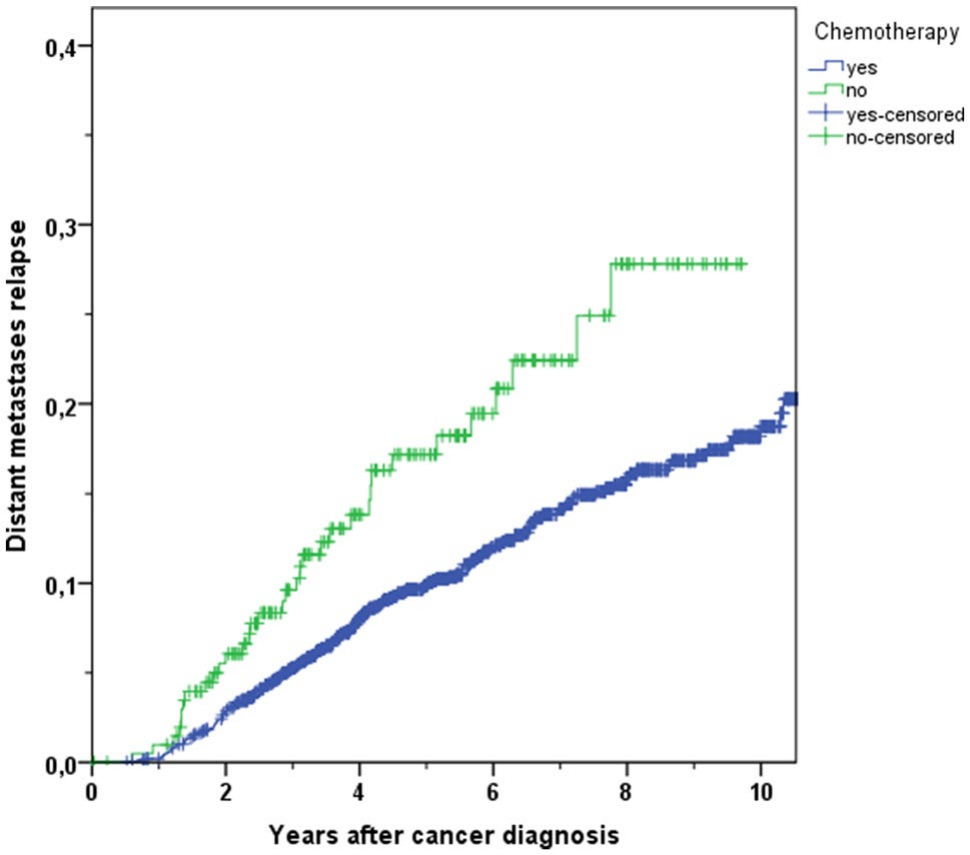
Kaplan–Meier plot of cumulative distant metastases recurrence rate in breast cancer patients with use or with non-use of chemotherapy

In the following, the distant metastases recurrence rate describes the frequency of distant metastatic lesions, which were recorded in our study. There was a significant benefit in using CHT evaluating both the 3-year distant metastases recurrence rate (5.4% in CHT-treated patients vs. 9.6% in not-treated patients) and the 5-year distant metastases recurrence rate (9.8% vs. 17.2%, *p* = 0.001, Fig. 3). Adjusted to all variables by means of multivariable Cox regression model analysis a significant lower recurrence rate persisted in patients obtaining CHT (HR 0.433, 95% CI 0.281–0.666, *p* < 0.001).

**Fig. 4:**
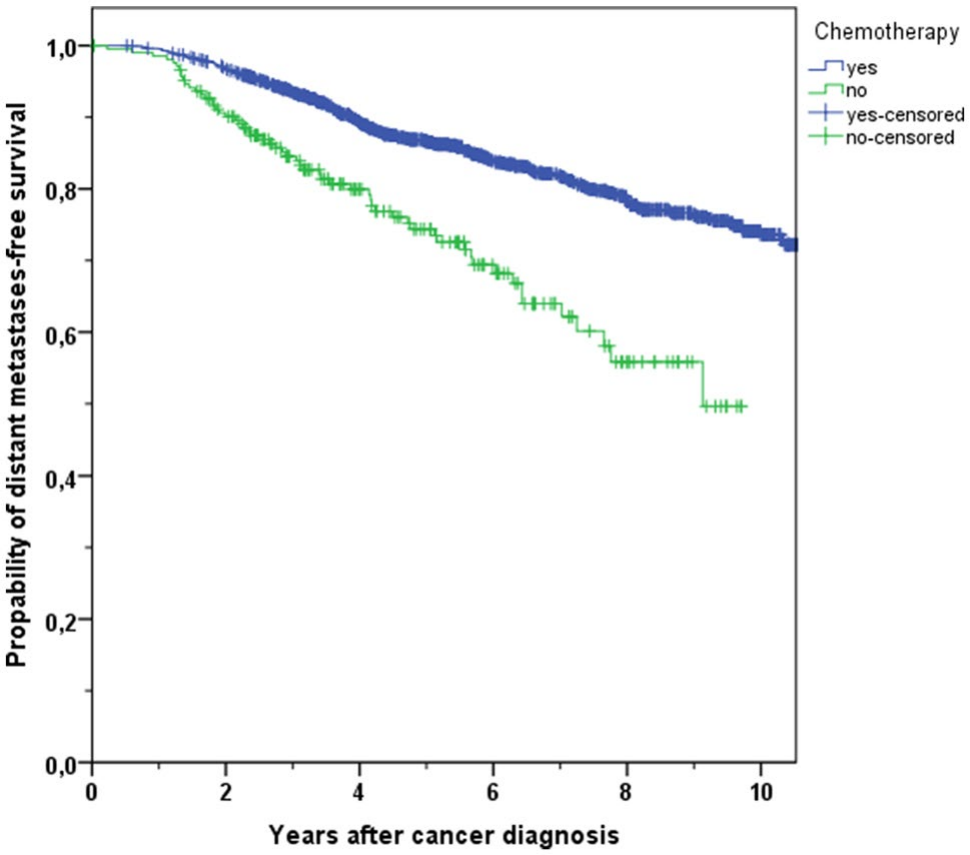
Kaplan–Meier plot of distant metastases-free survival in breast cancer patients with use or with non-use of chemotherapy

The distant metastases-free survival comprises the period after cancer diagnosis until distant metastases were detected. The 3-year and 5-year cumulative metastases-free survival rates in breast cancer patients with CHT were higher than in patients without treatment (93.4% vs. 84.5%, 86.7% vs. 74.4%, respectively, *p* < 0.001, Fig. 4). A multivariable Cox regression analysis confirmed the significant better DMFS in patients obtaining CHT (HR 0.484, 95% CI 0.344–0.682, *p* < 0.001).

### Subgroup analysis in Her2-negative patients

An additional analysis comprising a cohort with HER2-negative patients only (*N* = 1451 instead of *N* = 1772) showed no differences in OS and DMFS compared to the original data pool when comparing chemotherapy and no adjuvant treatment. The overall survival rates in patients with chemotherapy were 96.7%, 91.5%, and 76.2% after 3, 5 and 10 years compared with 90.9%, 81.0% and 47.0% in patients without chemotherapy. Multivariable analysis yielded a HR for OS of 0.543 (95% CI 0.361–0.816, *p* = 0.003). The DMFS rates in patients with CHT treatment were 93.9%, 87.3% and 73.2 after 3, 5 and 10 years vs. 87.2%, 77.2%, and 50.4% in patients without chemotherapy. Here, a HR of 0.548 was estimated (95% CI 0.373–0.804, *p* = 0.002).

### Effect of chemotherapy in two age groups

To differentiate the effect of CHT on overall and distant metastases-free survival, two age groups were compared in the complete cohort. Patients with age at diagnosis under 70 and patients being 70 years of age or older.

The first group with patients aged < 70 years included 1446 persons with 1354 obtaining (93.6%) vs. 92 not obtaining (6.4%) CHT. In contrast, the second group with 326 patients being 70 years of age or older obtained CHT more rarely. Of these, 190 patients (58.3%) received CHT.

**Fig. 5:**
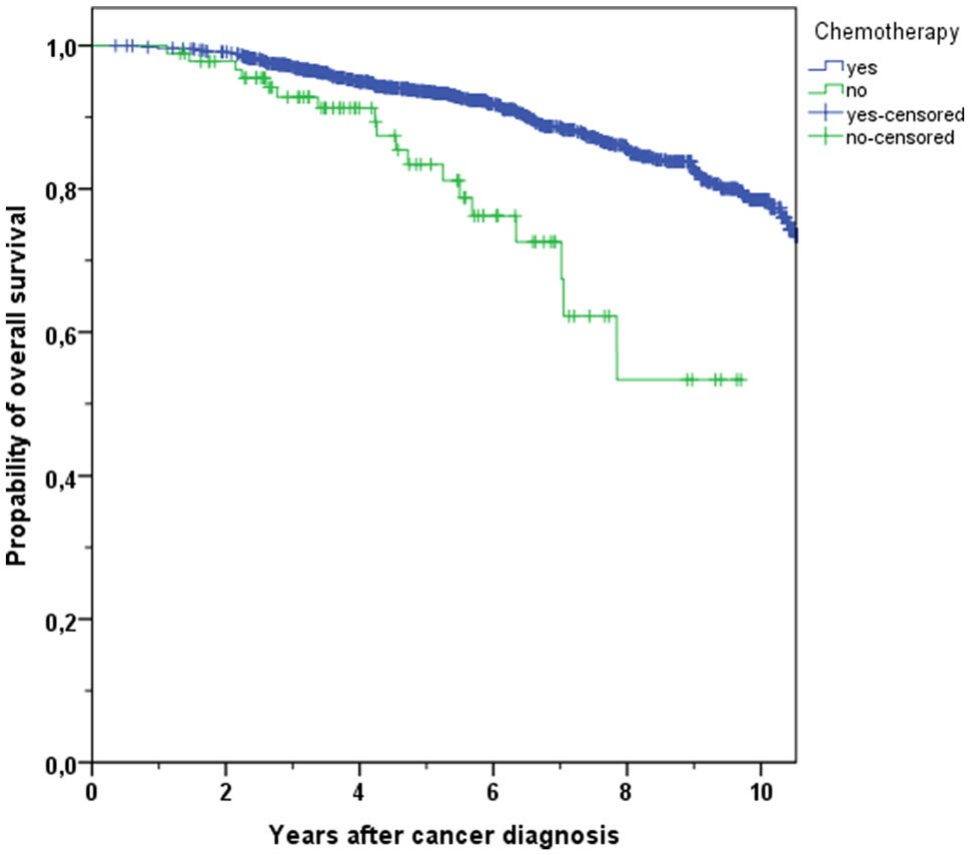
Kaplan–Meier plot of overall survival in breast cancer patients with age < 70 and with use and non-use of chemotherapy

In the group representing patients aging less than 70 (*N* = 1446), 182 persons passed away during the period of observation (12.1% of patients with CHT vs. 19.6% of patients without CHT). In the course of the years, there was a significantly better OS in patients receiving CHT (3-year OS 97.0% in treated patients vs. 92.8% in not-treated patients, 5-year OS 93.6% vs. 83.4%, *p* < 0.001, Fig. 5). Using Cox regression model, the better OS observed in the CHT group was confirmed (HR 0.270, 95% CI 0.161–0.451, *p* < 0.001, Table 4).

**Table 4:** Overview of overall survival (OS) and distant metastases-free survival (DMFS) rates in breast cancer patients after 3 and 5 years in different age groups

Age group and outcome	Chemotherapy	*N* Total	*N* Events	3 years (%)	5 years (%)	*p*
All age OS	Yes	1544	219	96.3	91.3	< 0.001
	No	228	64	88.7	76.8	
All age DMFS	Yes	1488	260	93.4	86.7	< 0.001
	No	207	58	84.5	74.4	
Age < 70 OS	Yes	1354	164	97.0	93.6	< 0.001
	No	92	18	92.8	83.4	
Age < 70 DMFS	Yes	1309	208	94.3	88.4	0.003
	No	87	19	87.7	78.6	
Age ≥ 70 OS	Yes	190	55	91.2	75.4	0.038
	No	136	46	85.9	72.6	
Age ≥ 70 DMFS	Yes	179	52	87.1	74.0	0.112
	No	120	39	82.3	71.4	

**Fig. 6:**
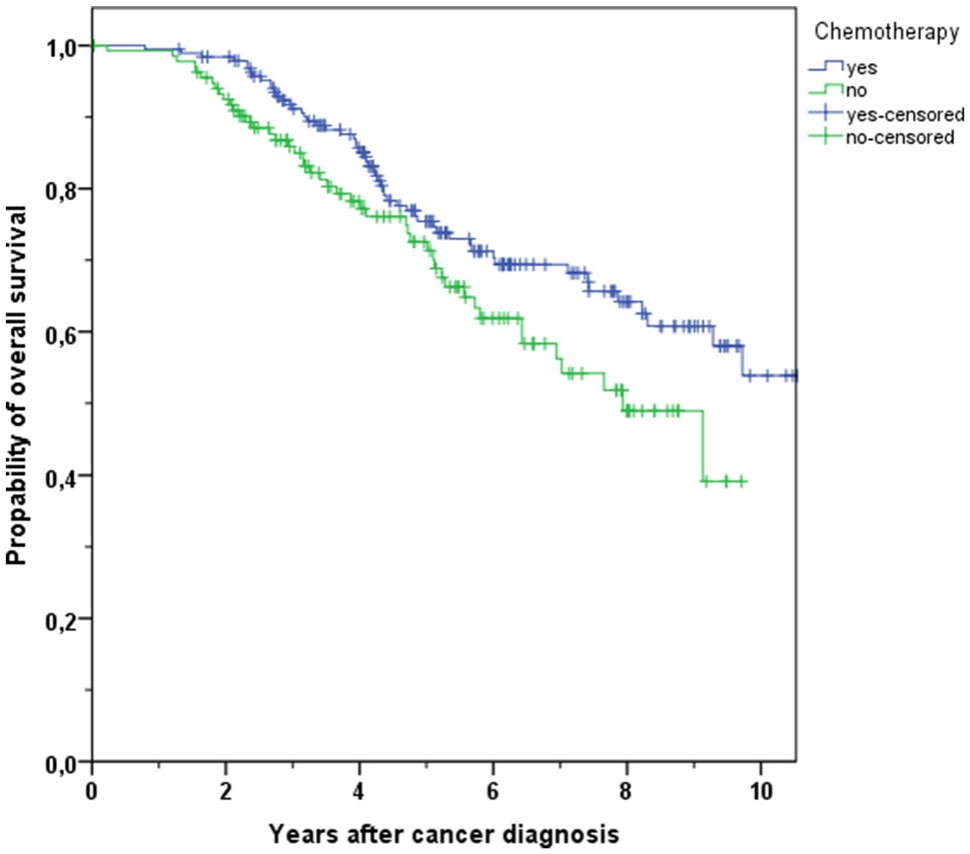
Kaplan–Meier plot of overall survival in breast cancer patients with age ≥ 70 and with use and non-use of chemotherapy

In contrast to this, the second group including the elderly patients with age ≥ 70 years (*N* = 326), 101 (31.0%) persons died in the follow-up time (28.9% of patients with CHT vs. 33.8% of patients without CHT). There was a small, but still significant OS benefit for the treated group (3-year OS 91.2% in treated patients vs. 85.9% in not-treated patients, 5-year OS 75.4% vs. 72.6%, *p* = 0.038, Fig. 6). However, a multivariable Cox regression model showed no significant benefit for patients with age 70+ and CHT treatment vs. no treatment (HR 0.754, 95% CI 0.470–1.209, *p* = 0.242).

**Fig. 7:**
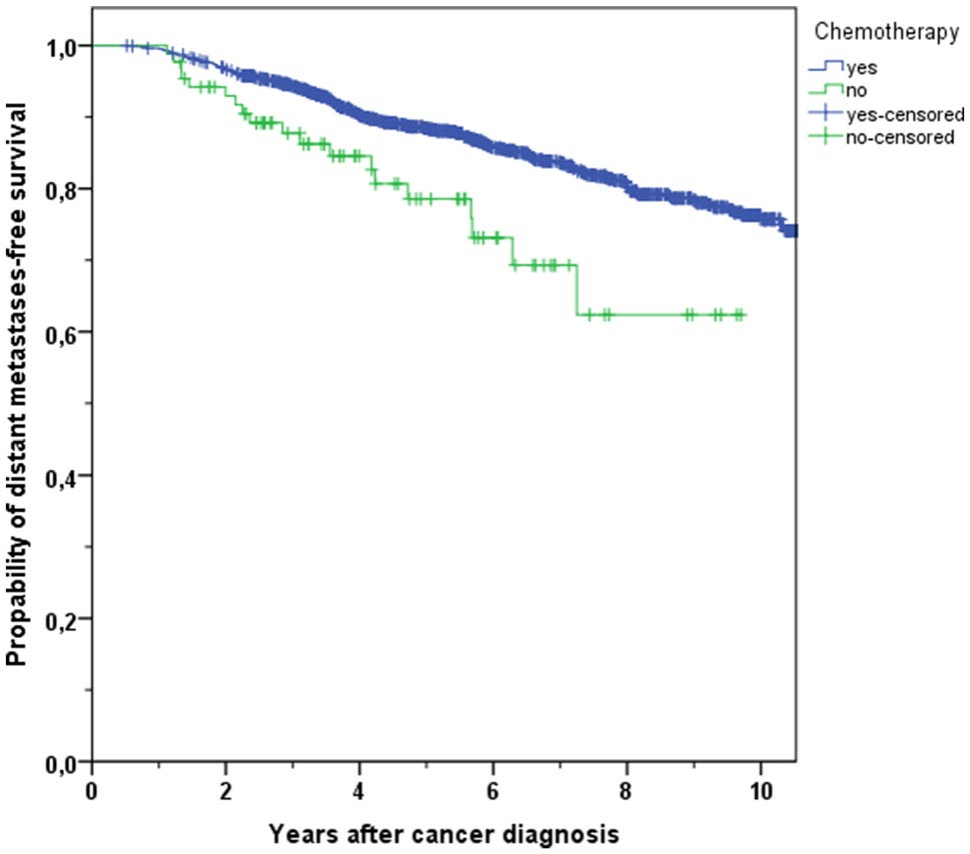
Kaplan–Meier plot of distant metastases-free survival in breast cancer patients with age < 70 and with use and non-use of chemotherapy

In addition to OS, the distant metastases-free survival was analysed. The first group representing the younger patients with R0 resection (*N* = 1396) listed 227 distant metastases relapses or deaths (15.9% of patients with CHT treatment vs. 21.8% of patients without treatment). In the younger patients’ group, the difference concerning the DMFS between the treatment and no treatment of CHT was significant (3-year DMFS 94.3% vs. 87.7% and 5-year DMFS 88.4% vs. 78.6%, *p* = 0.003, Fig. 7). A Cox regression model provided further evidence for a better DMFS in patients treated with CHT (HR 0.344, 95% CI 0.210–0.562, *p* < 0.001).

**Fig. 8:**
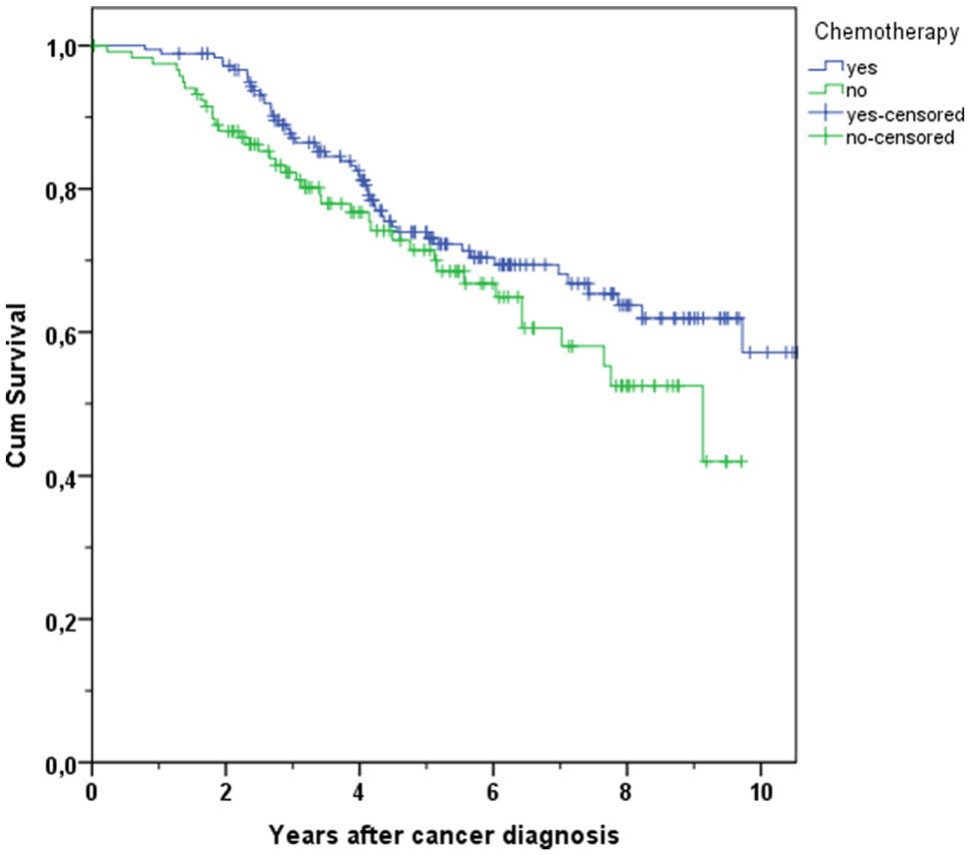
Kaplan–Meier plot of distant metastases-free survival in breast cancer patients with age ≥ 70 and with use and non-use of chemotherapy

In the group including patients aged 70 years or more and R0 resection (*N* = 299), 91 distant metastases relapses or deaths were noticed (29.1% of patients with chemotherapy treatment vs. 32.5% of patients without treatment). In the elder patients’ group, there was no significant difference concerning the DMFS between the treatment and no treatment of CHT (3-year DMFS 87.1% vs. 82.3% and 5-year DMFS 74.0% vs. 71.4%, *p* = 0.112, Fig. 8). Also, the Cox regression model showed no significant advantage in CHT treatment regarding the DMFS (HR 0.650, 95% CI 0.412–1.027, *p* = 0.065).

### Survival analyses based on different subgroups

To examine the potential effect modification of CHT on survival, the OS was analysed with multivariable Cox regression model in different subgroups divided into age at diagnosis, molecular subtypes (Luminal A, Luminal B) and nodal status (N1, N2/3). Across all age groups, CHT treatment in patients with Luminal A tumors leads to a significant better OS (HR 0.191, 95% CI 0.089–0.409, *p* < 0.001, Table 5), while Luminal B patients do only marginally benefit from CHT (HR 0.495, 95% CI 0.241–1.019, *p* = 0.056). Patients with low nodal status N1 as well as high nodal status N2/3 live significantly longer when treated with CHT (*p* = 0.001 and *p* = 0.038, respectively). Patients aged < 50 years with Luminal A tumor shows a significant better OS when treated with CHT, while young patients with low nodal status had no benefit. The analyses of CHT treatment in Luminal B patients and high nodal status tumors produce no statement about OS due to small number of events in this age group.

**Table 5:** Results of multivariable Cox proportional hazard model on overall survival based on subtype in patients of different ages

Age group	Subtype	*N* (CHT yes/no)	Hazard ratio (CHT yes/no)	95% CI	*p* value
All ages	Luminal A	540 (417/123)	0.191	0.089–0.409	< 0.001
	Luminal B	506 (455/51)	0.495	0.241–1.019	0.056
	N1	1077 (916/161)	0.394	0.229–0.680	0.001
	N2/3	695 (628/67)	0.585	0.352–0.972	0.038
Age < 50	Luminal A	149 (132/17)	0.053	0.006–0.500	0.010
	Luminal B	158 (155/3)	–^a^	–	0.968
	N1	328 (308/20)	0.211	0.041–1.091	0.063
	N2/3	192 (191/1)	–^a^	–	0.987
Age 50–69	Luminal A	288 (245/43)	0.166	0.053–0.520	0.002
	Luminal B	256 (243/13)	0.264	0.076–0.922	0.037
	N1	573 (518/55)	0.314	0.128–0.771	0.012
	N2/3	353 (337/16)	0.253	0.121–0527	< 0.001
Age ≥ 70	Luminal A	103 (40/63)	0.363	0.116–1.135	0.081
	Luminal B	92 (57/35)	0.496	0.203–1.212	0.124
	N1	176 (90/86)	0.459	0.222–0.951	0.036
	N2/3	150 (100/50)	1.091	0.561–2.125	0.797

^a^No estimate due to small number (coefficients did not converge)

In the middle-age group representing patients aged 50–69 years, CHT causes a survival benefit in both Luminal A and Luminal B patients. Likewise, better OS was seen in both treated groups with low and high nodal status. Contrary to this, neither Luminal A nor Luminal B patients with age ≥ 70 have better OS when treated with CHT in contrast to patients without treatment.

## Discussion

The implementation of the interdisciplinary S3 guideline of diagnosis, treatment and aftercare of breast cancer becomes established in the last 15 years. Regarding the certified breast cancer centers in Germany, which treated 78.8% of all patients in 2015 [9], the standardization of the breast cancer treatment results in an improvement and quality assurance of breast cancer care. Since the first certification of a breast cancer center in 2003, the number of these centers in Germany increase steadily to 266 in 2019 [10]. Patients treated in certified breast cancer centers can expect high-quality standards due to implementation of the national S3 guideline. Regarding systemic therapy, the guideline favours chemotherapy for patients with hormone receptor positive and node positive, invasive breast cancer. This study investigated implementation and effects of guideline concordant chemotherapy and demonstrates the long-term outcome of breast cancer patients based on data from a high-quality population-based regional cancer registry. In addition, it is worth analyzing elderly patients being 70 years of age or older in relation to younger patients separately. There is a lack of evidence for breast cancer care in elderly patients, because age is often a reason for exclusion from randomized clinical trials of breast cancer treatment [7].

During the period of observation, 87.1% of all patients with hormone positive and node positive, invasive breast cancer received chemotherapy in this study. This rate is comparable to other studies, the percentage of all hormone- and node-positive breast cancer patients treated with chemotherapy in 2010 being 89.7% [11]. So, data of this population-based regional cancer registry can be considered as representative for the health care in Germany and corresponds with the breast cancer care all over the country. Conspicuous is the decline of the treated patients regarding the 10 years of observation. This phenomenon is partly attributed to the improved documentation of the conducted therapies during the years. In the beginning of the observation, some of the breast cancer centers were just certified and the documentation was not as representative. With the improvement of the documentation, some patients who were first classified as “unknown therapy” and were excluded from analysis, were categorized in “no chemotherapy” in the course of the time. Additionally, changes in treatment standards are another reason for decreasing chemotherapy treatment in the end of the recruitment period. As Haque et al. describe, chemotherapy was used more rarely in Luminal A patients with low nodal status N1 in the course of time [12].

The age distribution showed that more than half of the patients with chemotherapy treatment were among 50 and 69 years old. Likewise, Inwald et al. confirm that chemotherapy and endocrine therapy is more often used in patients aging 50–69 than in patients being 70 years of age or older [13]. In addition, more than half of the patients not treated with chemotherapy were 70 years or older in our study. Reasons for the undertreatment in elderly patients are refusal of the patients themselves or recommendation of the attending physicians because of comorbidities or threatening negative side effects. The tumor characteristics between the treated and not-treated group differed in particular concerning the grading and the nodal status. Patients without chemotherapy treatment had more often a lower nodal status and a lower grading type. This trend may reflect the implementation of the S3 guideline, which claims for chemotherapy treatment in high-risk tumor types [3].

The survival benefit using chemotherapy vs. no treatment is evident regarding our study just as the 2011 EBCTCG polychemotherapy overview [14]. Palmieri et al. describes a reduction of 10-year breast cancer mortality by about a third in treated vs. not treated patients. This phenomenon is similar to the improvement of 5-year overall survival of 14.5% in our population-based study. Although this analysis includes only node-positive patients in contrast to all patients included in the polychemotherapy overview, the advantage of chemotherapy treatment is obvious.

A further study views the diverse ankles of chemotherapy treatment in node-positive breast cancer patients. Gnant et al. report on some subgroups of node-positive breast cancer patients with limited risk of metastasis. These patients should be spared from the negative side effects of chemotherapy treatment because of a too low benefit [15].

In our retrospective study, the different frequency of chemotherapy treatment between younger and elderly patients was remarkable (93.6% of patients aging < 70 were treated vs. 58.3% of patients aging ≥ 70). This tendency is in concordance with the SENORA project of the prospective German TMK (Tumour Registry Breast Cancer) cohort study, although it is worth mentioning, that patients with missing information about receiving CHT or not were not excluded. In the adjuvant setting of the SENORA project, 75.1% of all patients aging < 70 years received chemotherapy. In contrast to this, only 66.2% of patients aging ≥ 70 have undergone chemotherapy [16]. As mentioned above, age at diagnosis has a strong impact on the decision of chemotherapy treatment with a cautious attitude in elderly patients.

The evidence of better overall survival in chemotherapy-treated patients being < 70 years of age in comparison to not-treated patients is clearly shown in this analysis. According to studies, the survival benefit is indisputable, if there is a clear medical indication for chemotherapy treatment in younger breast cancer patients [17]. Therefore, chemotherapy is especially in younger patients without comorbidities an established treatment in breast cancer care for many years.

On the other hand, the study demonstrates that patients with age ≥ 70 made just a minimal profit from chemotherapy treatment concerning the overall survival in relation to not-treated patients the same age. Several studies analysed the conflicting use of chemotherapy in elderly patients with node-positive breast cancer with the same [18, 19] or differing conclusions [20]. In contrast to our analysis, Giordano et al. proved no benefit of chemotherapy among women with age ≥ 65 with lymph node-positive and estrogen receptor-positive breast cancer [21]. A lower risk of recurrence and death from other causes should be the reason for the missing advantage in elderly patients with node-positive and hormone receptor-positive breast cancer. On the other hand, the Early Breast Cancer Trialists’ Collaborative Group (EBCTCG) found that chemotherapy plus endocrine therapy in elderly patients is just minimally but still favourable, in contrast to major survival advantages in premenopausal patients [4]. Similar to this fact, Albain et al. confirmed the benefit of overall survival of chemotherapy combined with endocrine therapy vs. sole endocrine therapy in postmenopausal patients with node- and hormone receptor-positive breast cancer [22]. Apparently, the chemotherapy treatment in older patients with node-positive breast cancer is controversial and requires further investigation.

Clinical studies confirm the benefit of chemotherapy treatment in young patients with hormone receptor-positive and node-positive breast cancer exactly as our retrospective cohort study. The use of chemotherapy in elderly patients remains a controversial issue, particularly because most randomized trials exclude patients older than 70 years.

It has to be considered that particular patients do not benefit from CHT. Several studies describe no significant better OS for Luminal A patients with lymph node involvement treated with CHT [23, 24]. Likewise, Nielsen et al. describe that CHT treatment in premenopausal Luminal A patients results in no better OS [25]. In contrast to this, our analysis confirms the benefit of CHT in Luminal A patients being < 50 years. Consequently, CHT treatment in Luminal A patients remains a controversial issue.

Luminal B patients aged between 50 and 69, who represents the majority of all patients in our study, benefit from CHT treatment. With that, the recommendation of the St Gallen International Expert Consensus, which claims for CHT treatment in Luminal B patients, is confirmed in a population-based study [26]. Contrary to this, patients with age ≥ 70 and the same subtype do not show a better OS when treated with CHT according to our study. One option to resolve the conflict could be to take the Ki-67 score into consideration. Criscitiello et al. claim that Ki-67 expression identifies a subset of patients with Luminal B and node-positive breast cancer who could benefit from addition of CHT to endocrine therapy [27].

A limitation of our study is the missing information concerning non-oncologic comorbidities. This is a very important limitation of this survey, since patients with comorbidities are more likely to die from strenuous treatment side effects and, therefore, are not selected for chemotherapy. However, adjustment for age partially includes adjustment for comorbidities as a study has shown [28]. The older a patient is, the more non-oncologic comorbidities he suffers from. Furthermore, a survey of the Dutch Cancer Registry on colorectal cancer patients reported a significant association between age and the number of a person’s comorbidities [29]. Still, it would be desirable to have comorbidity score included in retrospective cohort analysis to be able to conduct an even more accurate risk adjustment.

In conclusion, patients with hormone-positive and node-positive, invasive breast cancer mainly benefit from CHT treatment. Nevertheless, there is a small fraction of these patients, where CHT is inadvisable.
